# Prevalence of enterotoxigenic *Bacteroides fragilis* in patients with colorectal cancer: a systematic review and meta-analysis

**DOI:** 10.3389/fcimb.2025.1525609

**Published:** 2025-03-07

**Authors:** Shijun Xia, Lijuan Ma, Hui Li, Yue Li, Linchong Yu

**Affiliations:** ^1^ Department of Anus & Intestine Surgery, Shenzhen Hospital (Fu Tian) of Guangzhou University of Chinese Medicine, Shenzhen, China; ^2^ Department of Anus & Intestine Surgery, Shenzhen Traditional Chinese Medicine Anorectal Hospital (Fu tian), Shenzhen, China; ^3^ State Key Laboratory of Traditional Chinese Medicine Syndrome/Research Group of Standardization of Chinese Medicine, The Second Affiliated Hospital of Guangzhou University of Chinese Medicine (Guangdong Provincial Hospital of Chinese Medicine), Guangzhou, China

**Keywords:** enterotoxigenic *Bacteroides fragilis*, colorectal cancer, prevalence, systematic review, meta-analysis

## Abstract

**Introduction:**

The gut microbiome, specifically enterotoxigenic *Bacteroides fragilis* (ETBF), has been reported to play a role in colorectal cancer development. We aimed to conduct a systematic review and meta-analysis of published studies to compare the prevalence of ETBF in patients with colorectal cancer and healthy controls as well as in various stages of colorectal cancer.

**Methods:**

PubMed, EMBASE, and The Cochrane Library were systematically searched for studies published until May 2024. We utilized studies either comparing the prevalence of ETBF in patients with colorectal cancer and healthy control or examining its prevalence across different stages of colorectal cancer. The prevalence of ETBF colonization in biological samples from individuals with colorectal cancer compared to that in healthy controls or adjacent normal tissue as well as the association between the prevalence of ETBF and various stages of colorectal cancer were plotted using a random-effect or fixed-effect model.

**Results:**

Fourteen relevant articles were identified. Meta-analyses revealed that patients with colorectal cancer had a higher likelihood of having ETBF than healthy controls (odds ratio [OR]: 2.54, 95% confidence interval [CI]: 1.63–3.98, I^2^ = 55%). Additionally, ETBF detection was lower in stage I/II than in stage III/IV colorectal cancer (OR: 0.61, 95% CI: 0.41–0.91, I^2^ = 41%).

**Discussion:**

The prevalence of ETBF was consistently higher in the tissue and fecal samples of patients with colorectal cancer than in those of controls. A difference in ETBF prevalence between stage I/II and stage III/IV colorectal cancer was noted, but further analysis revealed that the conclusion is unreliable.

**Systematic review registration:**

https://www.crd.york.ac.uk/prospero/, identifier CRD 42024548325.

## Introduction

Colorectal cancer (CRC) is the third most commonly diagnosed cancer in both men and women and the second leading cause of cancer-related deaths worldwide (Sung et al., 2021). The vast majority of CRC cases (1.9 million cases per year) are sporadic and can be attributed to various environmental factors (Islami et al., 2018). Cancer incidence in the large intestine is estimated to be 12-fold higher than that in the small intestine, which has been partially attributed to the greater bacterial density in the large intestine (Sun and Kato, 2016). In addition to host genetic factors, the gut microbiota plays an important role in CRC. An imbalance in the normal intestinal microbiota can promote chronic inflammation and carcinogenic metabolite production, ultimately leading to neoplasia (Marchesi et al., 2011).

Several bacterial species, including *Helicobacter pylori*, *Escherichia coli*, *Bacteroides fragilis*, *Salmonella enterica*, and *Fusobacterium nucleatum*, have been implicated in the development of CRC (Sun and Kato, 2016). A meta-analysis revealed a consistent increase in the prevalence of *F. nucleatum* in the tissue and fecal samples of patients with CRC compared to controls. Moreover, a high abundance of *F. nucleatum* in colorectal tumors was associated with poorer overall survival (Gethings-Behncke et al., 2020).

The anaerobe *B. fragilis* is a colonic symbiote that prefers mucosal colonization and accounts for only a small proportion of fecal microbiota (approximately 0.5%–1%). There are two molecular subtypes, nontoxigenic *B. fragilis* (NTBF) and enterotoxigenic *B. fragilis* (ETBF). According to some studies, ETBF is associated with both colitis and CRC (Basset et al., 2004; Toprak et al., 2006a; Dadgar-Zankbar et al., 2023). A review summarizes existing evidence for the association between ETBF and CRC as well as the current state of knowledge about the molecular mechanisms by which the *B. fragilis* toxin (BFT) influences the etiology of CRC (Scott et al., 2022). However, despite the increasing research on the relationship between ETBF and CRC, its role in the development of colorectal cancer remains largely uncertain (Zamani et al., 2020; Oliero et al., 2022). To our knowledge, no systematic reviews with meta-analyses have fully investigated the potential role of ETBF in CRC development.

This systematic review and meta-analyses of the published scientific literature aimed to assess (1) the prevalence of ETBF colonization in biological samples from individuals with CRC compared to healthy controls or adjacent normal tissues and (2) the relationship between the prevalence of ETBF and various stages of CRC.

## Methods

### Protocol and guidance

This study was conducted based on the Preferred Reporting Items for Systematic Reviews and Meta-Analyses reporting guidelines (Liberati et al., 2009). The review protocol has been registered with PROSPERO (CRD 42024548325). The need for ethical approval or informed consent was waived in this study.

### Search strategy

Following recommendations of the Meta-analysis of Observational Studies in Epidemiology group (Stroup et al., 2000), we searched the following electronic databases for studies written in English from their inception until May 15, 2024: PubMed, Embase, and The Cochrane Library. The following search terms were used: (“colorectal” or “colon” or “rectal”) and (“*Bacteroides fragilis*” or “*B. fragilis*” or “enterotoxigenic *B. fragilis*” or “enterotoxigenic *Bacteroides fragilis*” or “ETBF”). The search strategy was implemented by combining index words with free text keywords. In addition, the reference lists in these articles were reviewed to include more comprehensive studies.

### Inclusion and exclusion criteria

Study selection was performed independently, in duplicate, by two reviewers (SJX, LJM), with discrepancies resolved by a third reviewer (YL), using two levels of study screening.

Inclusion criteria were as follows: (1) cohort studies, (2) human studies, (3) studies involving patients with CRC, and (3) studies reporting the prevalence of ETBF in any biological sample.

Exclusion criteria were as follows: (1) studies involving participants with malignancies other than CRC, (2) those only recruiting patients with *B. fragilis* but no ETBF, and (3) those that could not obtain or calculate relevant data.

Furthermore, if duplicate articles were derived from the same or overlapping patient population, only the most recent and/or complete one was included in the meta-analysis. When there were multiple groups of useful data in the same article, only the data from the group with the largest sample size was used for the analysis.

### Data extraction and quality assessment

Data extraction was conducted independently, by two reviewers (SJX, LCY), with discrepancies resolved by a third reviewer (HL). The data included were authors, year of publication, study location, study design, ETBF detection method, sample type (tissue or fecal), participant status (patients with CRC or healthy controls), number of samples, and prevalence of ETBF in each sample.

Study quality was assessed using the Newcastle Ottawa Scale. Our meta-analysis categorized the study quality as good (≥7 stars), fair (4–6 stars), or poor (<4 stars).

### Statistical analysis

Regarding the prevalence of ETBF, meta-analyses were used to determine the pooled odds ratios (ORs) (the definition is provided in the **Supplementary Data Sheet 1**) and corresponding 95% confidence intervals (CIs) of ETBF prevalence in tissue and fecal samples, respectively, using published ORs, proportions, or numbers.

Review Manager version 5.3 (North Cochrane Center, Cochrane Collaboration, London, UK) was used to analyze data. Based on I^2^ values (the definition is provided in the **Supplementary Data Sheet 1**), four categories of heterogeneity were established: no heterogeneity (I^2^ < 25%), low heterogeneity (25% ≤ I^2^ < 50%), moderate heterogeneity (50% ≤ I^2^ < 75%), and high heterogeneity (I^2^ ≥ 75%). When the I^2^ value was <50%, a fixed-effects model was used, while a random-effects model was used for I^2^ > 50%.

## Results

**Figure 1:**
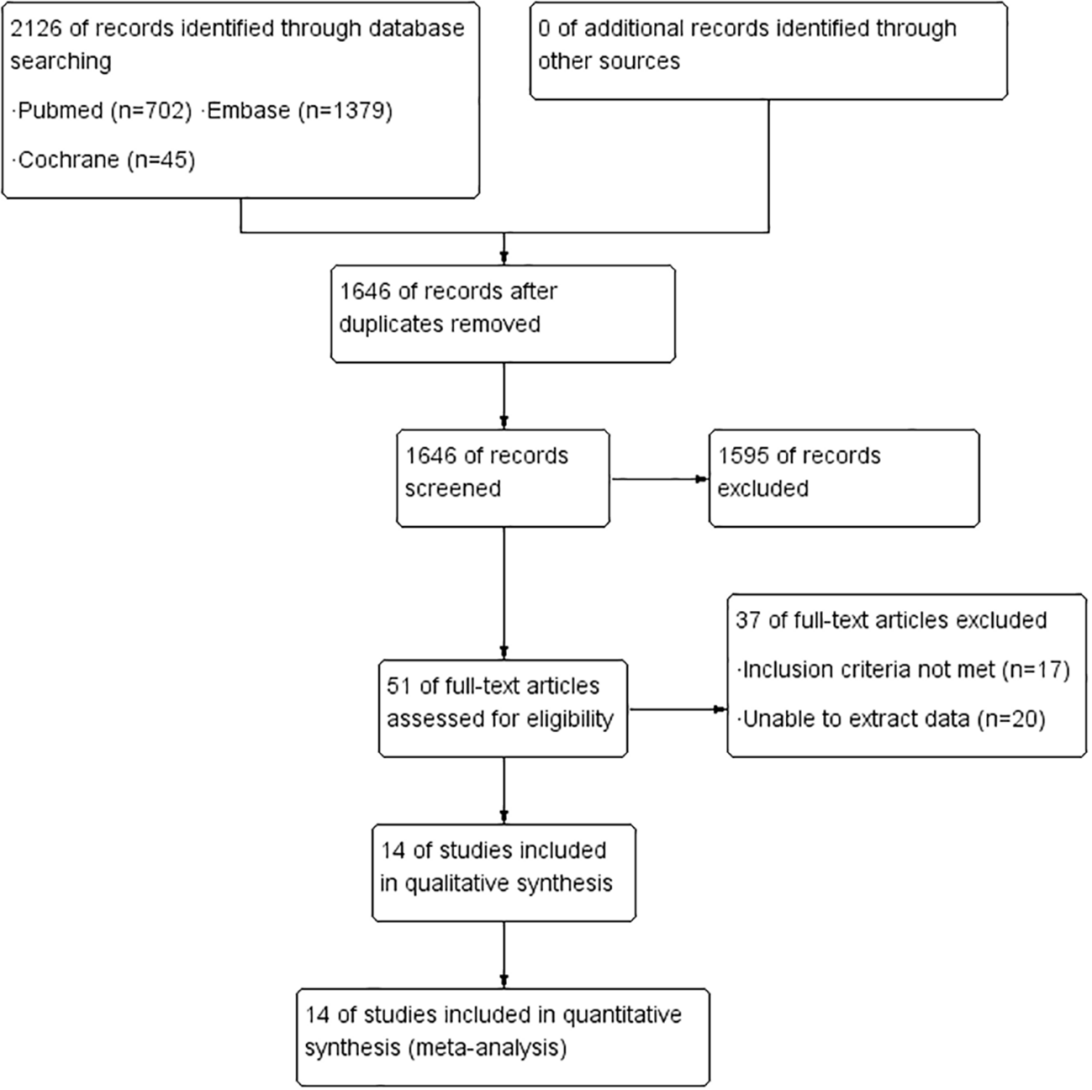
Flow chart of the identification of eligible studies.

After identifying 2126 references, we excluded 480 duplicate publications and 1595 irrelevant studies, leaving 51 potentially eligible studies (**Figure 1**). Finally, 14 cohort studies (Toprak et al., 2006b; Boleij et al., 2015; Viljoen et al., 2015; Keenan et al., 2016; Haghi et al., 2019; Jasemi et al., 2020; Zamani et al., 2020; Khodaverdi et al., 2021; Piciocchi et al., 2021; Shariati et al., 2021; Oliero et al., 2022; Périchon et al., 2022; Matsumiya et al., 2023; Zhou et al., 2023) conducted between 2006 and 2023 were considered for the meta-analysis. **Table 1** summarizes the general characteristics of the included studies. A total of 1692 patients were involved in these studies, with trial sizes ranging from 30 to 197 participants. Among these studies, two were from Europe, two from North America, six from West Asia, two from East Asia, one from South Africa, and one from New Zealand. Regarding the sample type, five studies used fecal samples, while nine used tissue samples. The detection method used was real-time polymerase chain reaction (PCR) in two studies, PCR in four studies, and quantitative PCR (qPCR) in eight studies. According to the quality assessment criteria, 11 studies were rated as good quality and 3 as fair quality.

**Table 1:** Characteristics of the included trials.

First Author	Country	Study type	Detection method	Sample type	Participant status	Tumor stage	Number of samples	Age/ years	Quality assessment
Toprak et al. (2006b)	Turkey	p	PCR	fecal	CRC vs healthy control	I-II-III	73 vs 59	63(24-90)	6
Boleij et al. (2015)	USA	P	PCR	tissue	CRC vs healthy control	I-II-III-IV	26 vs 37	62(52–76) /62(49–66)	6
Viljoen et al. (2015)	South Africa	R	qPCR	tissue	CRC	I-II-III-IV	54	59±15.3	6
Keenan et al. (2016)	New Zealand	R	qPCR	fecal	CRC vs healthy control	NR	71 vs 71	72(53-81) /64(53-80)	7
Haghi et al. (2019)	Iran	R	PCR	fecal	CRC vs healthy control	I-II-III	60 vs 60	53(29-90) /51(33-85)	7
Jasemi et al. (2020)	Iran	R	PCR	tissue	CRC vs healthy control	NR	31vs31	59.03±11.18 /57.35±10.79	7
Zamani et al. (2020)	Iran	R	Real-time PCR	tissue	CRC vs healthy control	NR	26vs52	55(35-78) /56(42-78)	7
Khodaverdi et al. (2021)	Iran	R	qPCR	tissue	CRC vs healthy control	I-II-III-IV	40vs40	56.37(31-86)/60(20-82)	7
Piciocchi et al. (2021)	Italy	R	qPCR	tissue	CRC vs healthy control	NR	29vs162	68(34-85) /59(22-87)	8
Shariati et al. (2021)	Iran	C	qPCR	tissue	CRC	I-II-III-IV	30	57±11.04	7
Oliero et al. (2022)	Canada	P	qPCR	fecal	CRC vs healthy control	I-II-III-IV	94vs62	67 [22–91] /58[24-78]	7
Périchon et al. (2022)	France	P	qPCR	fecal	CRC vs healthy control	I-II-III-IV	81vs25	63.5±7.9	8
Matsumiya et al. (2023)	Japan	R	Real-time PCR	tissue	CRC	II-III	197	70[40-91] /71[34-92]	8
Zhou et al. (2023)	China	R	qPCR	tissue	CRC	I-II-III-IV	92	64.60±14.50/65.80±10.40	8

R, retrospective study; P, prospective study; C, cross-sectional study; NR, not reported; CRC, colorectal cancer.

### Comparison between patients with CRC and healthy controls

**Figure 2:**
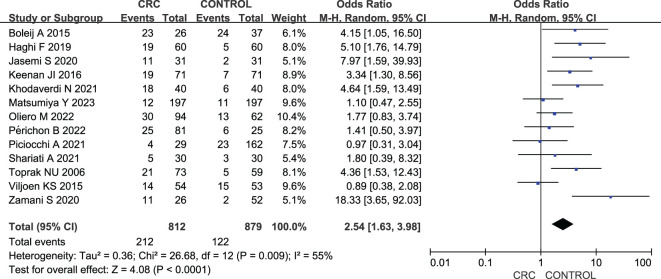
Forest plots of comparison between patients with colorectal cancer and healthy controls.

Thirteen studies examined the prevalence of ETBF in patients with CRC vs. healthy controls. As shown in **Figure 2**, a meta-analysis of ETBF prevalence indicated that the odds of ETBF detection were higher in patients with CRC than in healthy controls (OR: 2.54, 95% CI: 1.63–3.98, I^2^ = 55%).

Subgroup analyses were conducted based on country, sample type, and detection method (**Table 2**). A significant prevalence of ETBF was noted in both Europe, America, and Oceania region (OR: 1.95, 95% CI: 1.23–3.09, I^2^ = 7%) and West Asia region (OR: 5.09, 95% CI: 3.06–8.47, I^2^ = 0%); however, no difference in prevalence was noted between East Asia region (OR: 1.10, 95% CI: 0.47–2.55) and southern Africa region (OR: 0.89, 95% CI: 0.38–2.08). The results revealed significant associations in both colorectal tissue samples from separate individuals and fecal samples from separate individuals (OR: 4.42, 95% CI: 1.71–11.42, I^2^ = 60% and OR: 2.69, 95% CI: 1.67–4.35, I^2^ = 22%), but not in adjacent colorectal tissue samples [OR: 1.07, 95% CI: 1.61–1.87, I^2^ = 0%]. Regarding the detection method, the results showed a significant association in the use of both PCR and qPCR (OR: 4.95, 95% CI: 2.70–9.10, I^2^ = 0% and OR: 1.77, 95% CI: 1.13–2.79, I^2^ = 31%), but not in the use of real-time PCR (OR: 4.11, 95% CI: 0.26–65.83, I^2^ = 89%).

**Table 2:** Subgroup analysis of colorectal cancer compared to healthy controls.

Subgroup	No of study	Sample size	Heterogeneity I^2^	OR	P
Country					
Europe, America and Oceania	5	658	7%	1.95(1.23-3.09)	0.005
West Asia region	6	532	0%	5.09(3.06-8.47)	<0.00001
East Asia region	1	394		1.10(0.47-2.55)	0.83
Southern Africa region	1	107		0.89(0.38-2.08)	0.78
Sample type					
Colorectal tissue	5	474	60%	4.42(1.71-11.42)	0.002
fecal	5	656	22%	2.69(1.67-4.35)	<0.0001
adjacent colorectal tissue	3	561	0%	1.07(0.61-1.87)	0.81
Detection method					
PCR	4	377	0%	4.95(2.70-9.10)	<0.00001
qPCR	7	842	31%	1.77(1.13-2.79)	0.01
real-time PCR	2	472	89%	4.42(1.71-11.42)	0.32

### Comparison between stage I/II CRC and stage III/IV CRC

**Figure 3:**
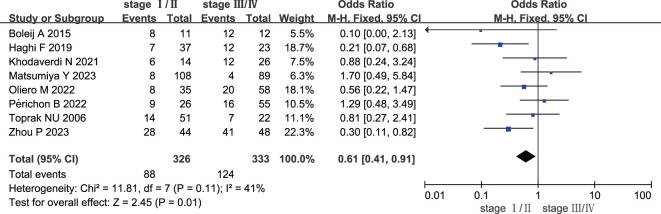
Forest plots of comparison between patients with stage I/II colorectal cancer and stage III/IV colorectal cancer.

Eight studies compared the prevalence of ETBF in **s**tage I/II CRC vs. stage III/IV CRC. As shown in **Figure 3**, a meta-analysis assessing ETBF prevalence revealed that the risk of ETBF being detected was lower in stage I/II CRC than in stage III/IV CRC (OR: 0.61, 95% CI: 0.41–0.91, I^2^ = 41%).

Subgroup analyses were also conducted based on country, sample type, and detection method (**Table 3**). There was no significant prevalence of ETBF in Europe (OR: 0.70, 95% CI: 0.27–1.81, I^2^ = 37%), West Asia region (OR: 0.53, 95% CI: 0.21–1.30, I^2^ = 43%), and East Asia region (OR: 0.69, 95% CI: 0.13–3.77, I^2^ = 78%). The findings revealed that the association was not significant in either colorectal tissue or fecal samples (OR: 0.59, 95% CI: 0.32–1.09, I^2^ = 52% and OR: 0.63, 95% CI: 0.38–1.05, I^2^ = 46%). The results of the detection method showed that there was no significant association in both the use of qPCR and real-time PCR (OR: 0.63, 95% CI: 0.38–1.06, I^2^ = 32% and OR: 1.70, 95% CI: 0.49–5.84), but there was a significant association with the use of PCR (OR: 0.38, 95% CI: 0.18–0.80, I^2^ = 44%).

**Table 3:** Subgroup analysis of stage I/II colorectal cancer compared to stage III/IV.

Subgroup	No of study	Sample size	Heterogeneity I^2^	OR	P
Country					
Europe region	3	197	37%	0.70(0.27-1.81)	0.47
West Asia region	3	173	43%	0.53(0.21-1.30)	0.16
East Asia region	2	289	78%	0.69(0.13-3.77)	0.66
Sample type					
Colorectal tissue	4	352	52%	0.59(0.32-1.09)	0.09
fecal	4	307	46%	0.63(0.38-1.05)	0.07
Detection method					
PCR	3	156	44%	0.38(0.18-0.80)	0.01
qPCR	4	306	32%	0.63(0.38-1.06)	0.08
real-time PCR	1	197	0	1.70(0.49-5.84)	0.40

### Sensitivity analysis

Sensitivity analysis was performed to assess the stability of the results, which resulted in the removal of one study from the meta-analysis at a time. The results revealed no change in the corresponding merged estimates of comparison between patients with colorectal cancer and healthy controls. **Table 4** presents the results of the sensitivity analysis. The results revealed a change in the corresponding merged estimates of comparison between patients with stage I/II colorectal cancer and stage III/IV colorectal cancer, indicating that two studies influenced the results: Haghi (2019) and Zhou (2023). Further investigation is required to elucidate the discrepancies between these two studies and the remaining six studies to ascertain the underlying causes responsible for this observed influence, which is beyond the scope of this work. **Table 5** presents the results of the sensitivity analysis.

**Table 4:** Sensitivity analysis results after removing one study at a time of comparison between patients with colorectal cancer and healthy controls.

Removed study	OR	95% CI	P	I2
Boleij et al. (2015)	2.47	1.54-3.96	0.0002	58%
Haghi et al. (2019)	2.39	1.50-3.81	0.0002	55%
Jasemi et al. (2020)	2.39	1.52-3.75	0.0002	55%
Keenan et al. (2016)	2.50	1.53-4.07	0.0002	58%
Khodaverdi et al. (2021)	2.42	1.51-3.88	0.0002	56%
Matsumiya et al. (2023)	2.78	1.74-4.43	<0.0001	53%
Oliero et al. (2022)	2.68	1.63-4.43	0.0001	58%
Périchon et al. (2022)	2.70	1.67-4.37	<0.0001	57%
Piciocchi et al. (2021)	2.75	1.73-4.37	<0.0001	55%
Shariati et al. (2021)	2.61	1.62-4.20	<0.0001	59%
Toprak et al. (2006b)	2.43	1.51-3.91	0.0002	56%
Viljoen et al. (2015)	2.82	1.80-4.40	<0.00001	48%
Zamani et al. (2020)	2.26	1.50-3.40	0.0001	45%

**Table 5:** Sensitivity analysis results after removing one study at a time of comparison between patients with stage I/II colorectal cancer and stage III/IV colorectal cancer.

Removed study	OR	95% CI	P	I^2^
Boleij et al. (2015)	0.64	0.43-0.96	0.03	42%
Haghi et al. (2019)	0.70	0.46-1.07	0.10	26%
Khodaverdi et al. (2021)	0.59	0.39-0.89	0.01	48%
Matsumiya et al. (2023)	0.54	0.35-0.82	0.004	33%
Oliero et al. (2022)	0.62	0.41-0.96	0.03	49%
Périchon et al. (2022)	0.53	0.35-0.82	0.004	36%
Toprak et al. (2006b)	0.59	0.39-0.90	0.01	48%
Zhou et al. (2023)	0.70	0.46-1.08	0.11	36%

### Publication bias

**Figure 4:**
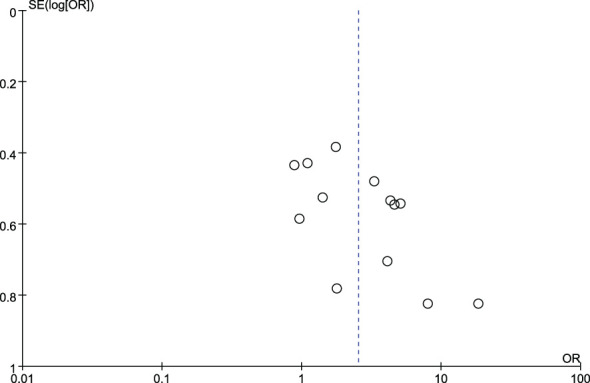
Funnel plot diagram of comparison between patients with colorectal cancer and healthy controls.

**Figure 5:**
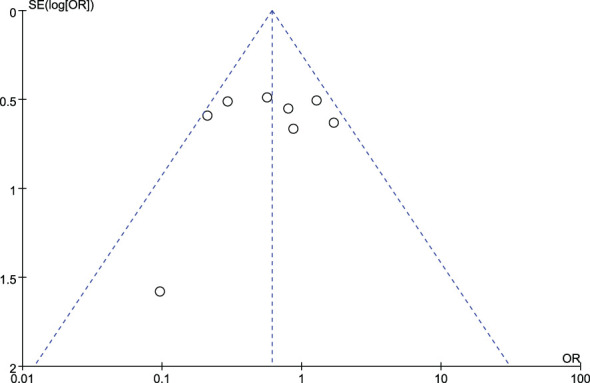
Funnel plot diagram of comparison between patients with stage I/II colorectal cancer and those with stage III/IV colorectal cancer.

**Figures 4** and **5** show funnel plots with scatter points that were generally symmetrical within the CIs, each study was evenly distributed on both sides of the vertical line, indicating that there was no significant publication bias.

## Discussion

The association between ETBF and CRC has attracted increasing interest. In this first comprehensive systematic review with meta-analyses of published literature, we aimed to investigate the relationship between ETBF and CRC, shedding light on its potential role in CRC development and progression.

Our findings suggest that ETBF is more prevalent in patients with CRC than in healthy controls, particularly in mucosal tissue or fecal samples from different individuals as shown by the subgroup analysis. The included studies revealed that ETBF colonization was more common in patients with CRC (6.1%–88.5%) than in healthy controls (3.8%–64.9%). This is consistent with previous research suggesting an association between ETBF colonization and CRC pathogenesis (Nouri et al., 2022). ETBF pathogenicity is attributed to BFT, a 20-kDa zinc-dependent metalloprotease toxin with three isotypes (BFT1, BFT-2, and BFT-3) (Sears, 2009). BFT binds to a specific colonic epithelial receptor, activating the Wnt and NF-κB signaling pathways, resulting in increased cell proliferation, epithelial release of proinflammatory mediators, and DNA damage (Sears, 2009; Goodwin et al., 2011), whereas ETBF promotes tumor formation in experimental animals (Wu et al., 2009; Goodwin et al., 2011).

Our analysis suggested that the detection rate of ETBF did not differ significantly between adjacent colorectal tissue samples and CRC tissue samples, but the next conclusion showed significant differences in ETBF prevalence between stage I/II and stage III/IV CRC. A previous study showed that ETBF supports the progression of malignancy as well as tumorigenesis (Kim and Lee, 2022). This suggests that ETBF may play a role in CRC initiation, and could possibly correlate with disease progression or severity. Therefore, this finding should be confirmed in larger cohorts.

Gut microbiota is a complex ecosystem that evolves in tandem with hosts and is influenced by their physiological environment. The composition and function of gut microbiota are closely associated with dietary habits and regional differences. Human dietary patterns have a direct impact on the abundance and diversity of gut microbiota. Diet is an important modifiable factor influencing the gut microbiome (Leeming et al., 2019). Furthermore, the proportion of plant-based and animal-based foods in the diet influences gut microbiota composition. The alteration in the abundance and diversity of gut microbiota caused by dietary changes has been associated with colorectal carcinogenesis (Appunni et al., 2021; Levy et al., 2021; Rebersek, 2021; Zygulska and Pierzchalski, 2022). Moreover, recent research has indicated that transitioning from a traditional to Western diet increases the abundance of CRC-associated bacteria (Ahmad Kendong et al., 2021). Another study demonstrated that switching from a traditional to Western diet increases the risk of CRC (Le Marchand and Kolonel, 1992). In our subgroup analysis of comparison between patients with CRC and healthy controls, the findings confirmed that individuals from different regions may have different outcomes due to differences in dietary habits.

These findings have two important implications. First, the increased prevalence of ETBF in the mucosal tissue or fecal samples of patients with CRC suggests that it can be used as a biomarker for CRC screening and diagnosis. Detection of ETBF may serve as an adjunctive tool in existing screening protocols to improve the sensitivity and specificity of CRC detection methods. Second, the consistent detection of ETBF in various stages of CRC highlights the need for additional research to determine its precise role in CRC pathogenesis. Understanding the mechanisms underlying ETBF-induced carcinogenesis may pave the way for targeted therapeutic interventions that disrupt the ETBF–CRC axis. However, a limitation is that the small number of studies prevented a formal assessment of publication or reporting bias, which may reduce the robustness of some meta-analyses involving subgroup analyses.

## Conclusions

There is consistent evidence that ETBF is more prevalent in the fecal and tissue samples of patients with CRC than in healthy controls. Further prospective studies into the role of ETBF as a causal factor or predictive biomarker for CRC promotion and development are warranted.

## Data Availability

The original contributions presented in the study are included in the article/**Supplementary Material**. Further inquiries can be directed to the corresponding author.

## References

[B1] Ahmad Kendong S. M. Raja Ali R. A. Nawawi K. N.M. Ahmad H. F. Mokhtar N. M. . (2021). Gut dysbiosis and intestinal barrier dysfunction: potential explanation for early-onset colorectal cancer. Front. Cell. Infect. Microbiol. 11, 1244. doi: 10.3389/fcimb.2021.744606 PMC8710575 34966694

[B2] Appunni S. Rubens M. Ramamoorthy V. Tonse R. Saxena A. McGranaghan P. . (2021). Emerging evidence on the effects of dietary factors on the gut microbiome in colorectal cancer. Front. Nutr. 8, 752. doi: 10.3389/fnut.2021.718389 PMC8542705 34708063

[B3] Basset C. Holton J. Bazeos A. Vaira D. Bloom S. (2004). Are Helicobacter species and enterotoxigenic Bacteroides fragilis involved in inflammatory bowel disease? Dig. Dis. Sci. 49, 1425–1432. doi: 10.1023/b:ddas.0000042241.13489.88 15481314

[B4] Boleij A. Hechenbleikner E. M. Goodwin A. C. Badani R. Stein E. M. Lazarev M. G. . (2015). The Bacteroides fragilis toxin gene is prevalent in the colon mucosa of colorectal cancer patients. Clin. Infect. Dis. 60, 208–215. doi: 10.1093/cid/ciu787 25305284 PMC4351371

[B5] Dadgar-Zankbar L. Shariati A. Bostanghadiri N. Elahi Z. Mirkalantari S. Razavi S. . (2023). Evaluation of enterotoxigenic Bacteroides fragilis correlation with the expression of cellular signaling pathway genes in Iranian patients with colorectal cancer. Infect. Agent Cancer 18, 48. doi: 10.1186/s13027-023-00523-w 37644520 PMC10463534

[B6] Gethings-Behncke C. Coleman H. G. Jordao H. W. T. Longley D. B. Crawford N. Murray L. J. . (2020). Fusobacterium nucleatum in the colorectum and its association with cancer risk and survival: A systematic review and meta-analysis. Cancer Epidemiol. Biomarkers Prev. 29, 539–548. doi: 10.1158/1055-9965.EPI-18-1295 31915144

[B7] Goodwin A. C. Shields C. E. D. Wu S. Huso D. L. Wu X. Murray-Stewart T. R. . (2011). Polyamine catabolism contributes to enterotoxigenic Bacteroides fragilis-induced colon tumorigenesis. Proc. Natl. Acad. Sci. 108, 15354e15359. doi: 10.1073/pnas.1010203108 21876161 PMC3174648

[B8] Haghi F. Goli E. Mirzaei B. Zeighami H. (2019). The association between fecal enterotoxigenic B. fragilis with colorectal cancer. BMC Cancer 19, 879. doi: 10.1186/s12885-019-6115-1 31488085 PMC6727388

[B9] Islami F. Goding Sauer A. Miller K. D. Siegel R. L. Fedewa S. A. Jacobs E. J. . (2018). Proportion and number of cancer cases and deaths attributable to potentially modifiable risk factors in the United States. CA Cancer J. Clin. 68, 31–54. doi: 10.3322/caac.21440 29160902

[B10] Jasemi S. Emaneini M. Fazeli M. S. Ahmadinejad Z. Nomanpour B. Sadeghpour Heravi F. . (2020). Toxigenic and non-toxigenic patterns I, II and III and biofilm-forming ability in Bacteroides fragilis strains isolated from patients diagnosed with colorectal cancer. Gut Pathog. 12, 28. doi: 10.1186/s13099-020-00366-5 32518594 PMC7273666

[B11] Keenan J. I. Aitchison A. Purcell R. V. Greenlees R. Pearson J. F. Frizelle F. A. (2016). Screening for enterotoxigenic Bacteroides fragilis in stool samples. Anaerobe 40, 50–53. doi: 10.1016/j.anaerobe.2016.05.004 27166180

[B12] Khodaverdi N. Zeighami H. Jalilvand A. Haghi F. Hesami N. (2021). High frequency of enterotoxigenic Bacteroides fragilis and Enterococcus faecalis in the paraffin-embedded tissues of Iranian colorectal cancer patients. BMC Cancer 21, 1353. doi: 10.1186/s12885-021-09110-x 34937552 PMC8693489

[B13] Kim J. Lee H. K. (2022). Potential role of the gut microbiome in colorectal cancer progression. Front. Immunol. 12. doi: 10.3389/fimmu.2021.807648 PMC8777015 35069592

[B14] Leeming E. R. Johnson A. J. Spector T. D. Le Roy C. I. (2019). Effect of diet on the gut microbiota: rethinking intervention duration. Nutrients 11, 2862. doi: 10.3390/nu11122862 31766592 PMC6950569

[B15] Le Marchand L. Kolonel L. (1992). Cancer in Japanese migrants to Hawaii: interaction between genes and environment. Rev. d’epidemiologie sante publique 40, 425–430. 1287741

[B16] Levy B. T. Daly J. M. Xu Y. Crockett S. D. Hoffman R. M. Dawson J. D. . (2021). Comparative effectiveness of five fecal immunochemical tests using colonoscopy as the gold standard: study protocol. Contemp. Clin. Trials 106, 106430. doi: 10.1016/j.cct.2021.106430 33974994 PMC8227954

[B17] Liberati A. Altman D. G. Tetzlaff J. Mulrow C. Gøtzsche P. C. Ioannidis J. P. . (2009). The PRISMA statement for reporting systematic reviews and meta-analyses of studies that evaluate health care interventions: explanation and elaboration. PloS Med. 6,7, e1000100. doi: 10.1371/journal.pmed.1000100 19621070 PMC2707010

[B18] Marchesi J. R. Dutilh B. E. Hall N. Peters W. H. Roelofs R. Boleij A. . (2011). Towards the human colorectal cancer microbiome. PloS One 6, e20447. doi: 10.1371/journal.pone.0020447 21647227 PMC3101260

[B19] Matsumiya Y. Suenaga M. Ishikawa T. Kudo T. Nakagawa T. Okamoto K. . (2023). Clinical significance of Bacteroides fragilis as a potential prognostic factor in colorectal cancer. Anaerobe 84, 102784. doi: 10.1016/j.anaerobe.2023.102784 37806638

[B20] Nouri R. Hasani A. Asgharzadeh M. Sefidan F. Y. Hemmati F. Rezaee M. A. (2022). Roles of gut microbiota in colorectal carcinogenesis providing a perspective for early diagnosis and treatment. Curr. Pharm. Biotechnol. 23, 1569–1580. doi: 10.2174/1389201023666220307112413 35255786

[B21] Oliero M. Hajjar R. Cuisiniere T. Fragoso G. Calvé A. Dagbert F. . (2022). Prevalence of pks + bacteria and enterotoxigenic Bacteroides fragilis in patients with colorectal cancer. Gut Pathog. 14, 51. doi: 10.1186/s13099-022-00523-y 36578036 PMC9798702

[B22] Périchon B. Lichtl-Häfele J. Bergsten E. Delage V. Trieu-Cuot P. Sansonetti P. . (2022). Detection of Streptococcus gallolyticus and Four Other CRC-Associated Bacteria in Patient Stools Reveals a Potential “Driver” Role for Enterotoxigenic Bacteroides fragilis. Front. Cell Infect. Microbiol. 12. doi: 10.3389/fcimb.2022.794391 PMC8963412 35360109

[B23] Piciocchi A. Germinario E. A.P. Garcia Etxebarria K. Rossi S. Sanchez-Mete L. Porowska B. . (2021). Association of polygenic risk score and bacterial toxins at screening colonoscopy with colorectal cancer progression: A multicenter case-control study. Toxins (Basel). 13, 569. doi: 10.3390/toxins13080569 34437440 PMC8402601

[B24] Rebersek M. (2021). Gut microbiome and its role in colorectal cancer. BMC Cancer 21, 1–13. doi: 10.1186/s12885-021-09054-2 34895176 PMC8666072

[B25] Scott N. Whittle E. Jeraldo P. Chia N. (2022). A systemic review of the role of enterotoxic Bacteroides fragilis in colorectal cancer. Neoplasia 29, 100797. doi: 10.1016/j.neo.2022.100797 35461079 PMC9046963

[B26] Sears C. L. (2009). Enterotoxigenic Bacteroides fragilis: a rogue among symbiotes. Clin. Microbiol. Rev. 22, 349e369. doi: 10.1128/CMR.00053-08 19366918 PMC2668231

[B27] Shariati A. Razavi S. Ghaznavi-Rad E. Jahanbin B. Akbari A. Norzaee S. . (2021). Association between colorectal cancer and Fusobacterium nucleatum and Bacteroides fragilis bacteria in Iranian patients: a preliminary study. Infect. Agent Cancer 16, 41. doi: 10.1186/s13027-021-00381-4 34108031 PMC8191199

[B28] Stroup D. F. Berlin J. A. Morton S. C. Olkin I. Williamson G. D. Rennie D. . (2000). Meta-analysis of observational studies in epidemiology: a proposal for reporting. Meta-analysis Of Observational Studies in Epidemiology (MOOSE) group. JAMA 283, 2008–2012. doi: 10.1001/jama.283.15.2008 10789670

[B29] Sun J. Kato I. (2016). Gut microbiota, inflammation and colorectal cancer. Genes Dis. 3, 130–143. doi: 10.1016/j.gendis.2016.03.004 28078319 PMC5221561

[B30] Sung H. Ferlay J. Siegel R. L. Laversanne M. Soerjomataram I. Jemal A. . (2021). Global cancer statistics 2020: GLOBOCAN estimates of incidence and mortality worldwide for 36 cancers in 185 countries. CA Cancer J. Clin. 71, 209–249. doi: 10.3322/caac.21660 33538338

[B31] Toprak N. U. Yagci A. Gulluoglu B. M. Akin M. L. Demirkalem P. Celenk T. . (2006a). A possible role of Bacteroides fragilis enterotoxin in the aetiology of colorectal cancer. Clin. Microbiol. Infect. 12, 782–786. doi: 10.1111/j.1469-0691.2006.01494.x 16842574

[B32] Toprak N. U. Yagci A. Gulluoglu B. M. . (2006b). A possible role of Bacteroides fragilis enterotoxin in the aetiology of colorectal cancer. Clin. Microbiol. Infect. 12, 782–786. doi: 10.1111/j.1469-0691.2006.01494.x 16842574

[B33] Viljoen K. S. Dakshinamurthy A. Goldberg P. Blackburn J. M. (2015). Quantitative profiling of colorectal cancer-associated bacteria reveals associations between fusobacterium spp., enterotoxigenic Bacteroides fragilis (ETBF) and clinicopathological features of colorectal cancer. PloS One 10, e0119462. doi: 10.1371/journal.pone.0119462 25751261 PMC4353626

[B34] Wu S. Rhee K.-J. Albesiano E. Rhee K. J. Albesiano E. Rabizadeh S. Wu X. Yen H. R. . (2009). A human colonic commensal promotes colon tumorigenesis via activation of T helper type 17 T cell responses. Nat. Med. 15, 1016e1022. doi: 10.1038/nm.2015 19701202 PMC3034219

[B35] Zamani S. Taslimi R. Sarabi A. Jasemi S. Sechi L. A. Feizabadi M. M. (2020). Enterotoxigenic bacteroides fragilis: A possible etiological candidate for bacterially-induced colorectal precancerous and cancerous lesions. Front. Cell Infect. Microbiol. 9. doi: 10.3389/fcimb.2019.00449 PMC6978650 32010637

[B36] Zhou P. Dai Z. Xie Y. Li T. Xu Z. Huang Y. . (2023). Differences in tissue-associated bacteria between metastatic and non-metastatic colorectal cancer. Front. Microbiol. 14. doi: 10.3389/fmicb.2023.1133607 PMC10289161 37362927

[B37] Zygulska A. L. Pierzchalski P. (2022). Novel diagnostic biomarkers in colorectal cancer. Int. J. Mol. Sci. 23, 852.29. doi: 10.3390/ijms23020852 35055034 PMC8776048

