# Supplementary material for: Prevalence of enterotoxigenic Bacteroides fragilis in patients with colorectal cancer: a systematic review and meta-analysis
Source: Front Cell Infect Microbiol. 2025 Mar 7;15:1525609. doi: 10.3389/fcimb.2025.1525609 (PMC11926129; doi:10.3389/fcimb.2025.1525609)
Supplement: Supplementary file 1 [file DataSheet1.docx]

1. OR is the ratio of the number of exposed individuals to the number of non-exposed individuals in the case group, divided by the ratio of the number of exposed individuals to the number of non-exposed individuals in the control group.

2. I² is used in meta-analysis to measure the percentage of total variation in effect sizes that is due to heterogeneity rather than chance. Specifically, the I² value indicates the proportion of the observed variance that cannot be attributed to sampling error, reflecting the degree of overlap in the confidence intervals of individual study estimates.
